# “We just take care after each other”: Relational health strategies of nurses and nursing aides working in residential long-term care as a mechanism of in- and exclusion in care teams

**DOI:** 10.3233/WOR-220653

**Published:** 2024-08-06

**Authors:** Saskia Elise Duijs, Martine van Wees, Tineke Abma, Zohra Bourik, Olivia Plak, Yvonne Jaspers, Usha Jhingoeri, Naziha Senoussi, Petra Verdonk

**Affiliations:** a Department of Ethics, Law and Humanities, Amsterdam UMC, Amsterdam, The Netherlands; b Amsterdam Public Health Research Institute, Amsterdam, The Netherlands; c Leyden Academy on Vitality and Ageing, Leiden, The Netherlands

**Keywords:** Low-paid care workers, long-term care, occupational health, gender, intersectionality

## Abstract

**BACKGROUND:**

The health of care workers in residential long-term care (LTC) is under pressure. Scholars emphasize the importance of gender-sensitive and intersectional approaches to occupational health.

**OBJECTIVE:**

To unravel how the health of nurses and nursing aides is shaped by gender, class, age, sexuality and race.

**METHODS:**

A qualitative participatory study. A participatory research team, consisting of academic scholars and nursing aides, conducted semi-structures interviews (*N* = 20) and one natural group discussion (*N* = 8 participants) to validate our findings. Thematic data-analysis was guided by gender and intersectionality theory.

**RESULTS:**

Empirical findings suggest that gendered norms limit possibilities for self-care for female nurses and nursing aides. Feeling uncared for by society and LTC organizations, paid care workers describe how they take care of each other. These relational health strategies require a feeling of sameness, limiting space for diversity and disability within care teams. Care workers seen as ‘cultural other’, or those who experienced (chronic) health issues, financial struggles or informal caregiving, risked being excluded from relational care within care teams, which negatively impacted their health.

**CONCLUSIONS:**

Care workers’ relational health strategies are a gendered and care ethical response to the lack of societal and political care for LTC, but can become mechanisms of exclusion within care teams. This can be understood as a sign of exhaustion, shaped by adverse working conditions and leading to moral stress. The lack of societal appreciation needs to be addressed by occupational health physicians and LTC organizations, to counter mechanisms of exclusion among paid care workers.

## 1 Introduction

Occupational health scholars express concerns about the health and wellbeing of low-paid care workers in long-term care (LTC) [1, 2]. Low-paid care workers such as (advanced) nursing aides make up the majority of the workforce in LTC and their health appears to be most at risk, yet they largely remain invisible in occupational health literature [3].

Also in the Netherlands, occupational health concerns are specifically directed at (advanced) nursing aides in residential long-term care (LTC) for older people [4, 5]. Recent research shows that sickness absence of LTC workers is among the highest in the healthcare sector and growing [6], staff turnover is high [6, 7]. Moreover, many older care workers opt for early retirement [7], and about 40% of care workers intend to leave the sector within the next five years [8]. In the context of an ageing population with rapidly increasing care needs and staff shortages, it is especially urgent to protect, maintain and repair the health of care workers [9]. In the Netherlands, a strong urgency is felt by LTC organizations, professionals’ associations and unions to support the health and wellbeing of low-paid LTC workers and they collectively express their concerns [10].

The majority of LTC workers are women and a gender perspective is essential to understand their health experiences and strategies [11, 12]. In the Netherlands, about 80% of the health and social care workforce health are women [13]. This percentage is higher in low-paid caring occupations: 94% of the workers in LTC are women [13]. Men and women occupy different spaces and positions in labor markets, characterized by horizontal and vertical gender segregation [14]. Attention among occupational health scholars about sex-and gender specific occupational health issues is growing [12]. For women, this, for example, led to more knowledge about menopause and occupational health [15]. Research also indicates that percentages and social dynamics around sickness absence are distinctly gendered [16, 17], just as the work/life conflict, due to women’s larger responsibility for unpaid caring responsibilities compared to men [18]. As a consequence, women working in LTC have different health issues, health strategies and occupational health needs compared to men working in LTC. More knowledge is needed on how women working in LTC maintain, protect and repair their health.

In the Netherlands, concerns about the health of LTC workers became a pressing societal issue after major policy reforms in long-term care [19]. In the Netherlands, these policy reforms were installed in 2015. As a result of neoliberal political decision making, care responsibilities were allocated towards lower professionals’ levels, and from paid to unpaid caregivers [19]. This policy dramatically impacted the working conditions of LTC workers. Not only did it result in more complex care tasks for an increased number of clients, for those who provided informal care tasks it also led to more informal care responsibilities at home [21]. Early on, in 2013, studies already showed that these policy measures would particularly impact *women*’*s* work, health and informal care responsibilities, as women are the default caregivers [22]. In response, women’s organizations advocated for gender and diversity-sensitive perspectives on LTC workers’ occupational health [22, 23].

In occupational health, researchers are increasingly adopting intersectionality as a theoretical and methodological approach for gender and diversity sensitive research [24, 25]. An intersectional perspective explores how people’s lived experiences are shaped by multiple aspects of one’s identity and intersecting systems of inequity including gender, but also class, race sexuality, age, life phase [26, 27]. A gender-only perspective runs the risk of overlooking differences *among* women [28]. Intersectionality is rooted in black, feminist, queer, postcolonial, thought and activism and coined as a concept by Kimerblé Crenshaw [29]. Gender and intersectionality theory urge scholars to take the societal and organizational context into account, as health inequalities are (re-) produced within organizations, and within (care) teams [30–32].

This paper reports on the outcomes of a qualitative interview study [33, 34] that took place in the Netherlands (2019–2020). This study explores how the health and health strategies of nurses and (advanced) nursing aides in LTC for older people is shaped by gender in intersection with class, race, ability and age/life-phase. An intersectionality-informed approach was applied to understand differences and inequities among LTC workers, and to analyze how health inequalities are re-produced within LTC teams and organizations. By using intersectionality as a main framework for analysis, this paper aims to contribute to advance gender and diversity-sensitive occupational health care and interventions.

## 2 Methods

The consolidated criteria for reporting qualitative research (COREQ) guided the reporting of the study [35].

### 2.1 Research team and reflexivity

The participatory research team consisted of four Dutch-majority women and Moroccan-Dutch woman, working as academic researchers (SD, TA, ZB, PV). The academic researchers were experts in gender, intersectionality and (occupational) health (SD, PV), participation in LCT (TA), Islam, diversity and community health (ZB). The community researchers, four women and one man, from diverse cultural backgrounds, were experts by experience as paid and unpaid care workers, three of which were trained as advanced nursing aides. Interviews were conducted in a couple existing of an academic researcher (SD, ZB) with one of the community researchers (OP, UJ, YJ, NS).

### 2.2 Design

This participatory, qualitative interview study was a sub-study in a broader participatory health research project [36, 37]. This project “Negotiating Health” aimed to unravel how the health of paid and unpaid care workers in residential LTC was shaped by intersecting social determinants of health, such as gender, class, race, disability sexuality and age [24–32]. Participatory health research aims to understand how peoples’ lived experiences are shaped by structural inequalities, together with those whose life or work is subject to the study [38, 39].

While quantitative research has been traditionally dominant within occupational health research, researchers emphasized the added value of qualitative research [34]. Qualitative research aims to understand people’s experiences, behaviors and interactions, it can provide insights about new or relatively unknown phenomena and help to understand the larger social context that shapes peoples’ subjective experiences [33, 34].

Epistemologically, our research is grounded in phenomenology (focusing on lived and subjective experiences), critical theory (unraveling how these are shaped by structural inequalities, using critical theory such as intersectionality) and participatory approaches (not doing research ‘about, but doing research ‘with’ people) [33, 37].

In ‘Negotiating Health’, interviews were conducted with hired employees and self-employed care workers, men and women. This article focusses on the experiences of women working as hired employees within LTC organizations. Findings from other studies are published elsewhere [38, 41].

Participants of the current interview study were recruited through organizations (such as the professional associations for nurses and nursing aides, unions, LTC organizations, support organizations for informal caregivers), through social media as well through the personal network of community researchers. Participants were approached with a general invitation flyer. Inclusion criteria were: being an (advanced) nursing aide, identifying as women, 45–67 years of age, combing paid and unpaid care responsibilities. Eventually, 20 care workers were purposively sampled, also including some nurses, who were higher paid, as a deviant case. Participants came from diverse geographical locations in the Netherlands. As a result of our recruitment strategy, via mainstream channels and organizations, our sample largely consists of white, Dutch majority, women (see Table 1). All participants that responded to our flyer were included; hence our population can be characterized as an ‘convenience sample’ [33]. Interviews were conducted until reached data-saturation was reached within this particular population. Interviews were conducted in a duo of academic researcher (authors) and a community researcher (authors). Interview were mostly conducted at home with the participants. Some participants wished to be interviewed or at their workplace, in a public space or at the university. In two instances, the interview was conducted in presence of a colleague or partner. One interviewee [R17] was so supportive of our study that she proposed to organized a meeting in her home to discuss our preliminary findings with her and seven of her colleagues (total *N* = 8 participants). This natural group discussion [33] was facilitated by two academic researchers and a community researcher (author names). This meeting was audio-recorded and transcribed ad-verbatim.

**Table 1:** Participants interviews

R	Age	Minority/majority	Relationship status	Unpaid care responsibilities	Profession
1	66	Surinam-Dutch	Heterosexual marriage	Niece	Advanced nursing aide (level 3)
2	51	Surinam-Dutch	Heterosexual marriage	Mother and brother	Advanced nursing aide (level 3)
3	48	Dutch-Majority	Heterosexual marriage	Parents	Advanced nursing aide (level 3)
4	54	Dutch-Majority	Divorced (heterosexual marriage)	Mother[+] Father[+] Two close friends	Advanced nursing aide (level 3)
5	52	Dutch-Majority	Heterosexual marriage	Mother and parents-in-law	Advanced nursing aide (level 3)
6	45	Moroccan-Dutch	Heterosexual marriage	Mother	Nurse (level 6)
7	47	Dutch-Majority	Heterosexual marriage	Mother	Advanced nursing aide (level 3)
8	58	Dutch-Majority	Heterosexual marriage	Sister	Advanced nursing aide (level 3)
9	50	Dutch-Majority	Heterosexual marriage	Mother in law	Advanced nursing aide (level 3)
10	45	German-Dutch (visibly Muslim)	Heterosexual marriage, divorced	Father	Nursing aide (level 2)
11	61	Dutch-Majority	Heterosexual marriage	Husband	Advanced nursing aide (level 3)
12	45	Dutch-Majority	Heterosexual marriage	Husband	Advanced nursing aide (level 3)
13	53	Dutch-Majority	Heterosexual marriage	Husband	Advanced nursing aide (level 3)
14	60	Dutch-Majority	Heterosexual marriage	Father, Mother, Daughter	Advanced nursing aide (level 3)
15	52	Dutch -Majority	Heterosexual marriage	Mother[+]	Advanced nursing aide (level 3)
16	+/–55	Dutch-Majority	Heterosexual marriage	Father, Son	Advanced nursing aide (level 3)
17	62	Dutch-Majority	Heterosexual marriage, widowed	Husband	Advanced nursing aide (level 3)
18	57	Dutch-Majority	Heterosexual marriage	Godmother, mother	Nurse assistant (level 1)
19	52	Dutch-Majority	Heterosexual marriage	Children, mother, sister	Advanced nursing aide (level 3
20	45	Moroccan-Dutch	Heterosexual marriage	Parents	Nursing aide (level 2)

**Table 2:** Participants Natural Group Discussion

FG	Age	Gender	Minority/Majority	Profession
1	+/–50^*^	Woman	Dutch-Majority	Advanced nursing aide (level 3)
2	+/–55^*^	Woman	Dutch-Majority	Advanced nursing aide (level 3)
3	54	Woman	Dutch-Majority	Advanced nursing aide (level 3)
4	61	Woman	Dutch-Majority	Advanced nursing aide (level 3)
5	60	Woman	Dutch-Majority	Advanced nursing aide (level 3)
6	+/–55^*^	Woman	Dutch-Majority	Advanced nursing aide (level 3)
7	40	Woman	Dutch-Majority	Advanced nursing aide (level 3)

^*^Not everyone wanted to disclose their age during this natural group discussion. This is an estimation from the authors.

### 2.3 Data collection

Participants provided consent before participating. The topic list for this study was developed based on literature and by the community researchers who identified themes and topics that were relevant to them during a six-month photovoice project that was conducted prior to this qualitative study [38]. Themes included balancing paid and unpaid care work, financial concerns, age-related health issues, such as menopause, and health strategies of care workers while working in LTC (Appendix #1 and #2). Interviews are recorded and transcribed ad verbatim. Field notes were made by both interviewers separately, after the interview. Interviews were conducted in Dutch. Quotes in this article are translated by the first author. Member checks were conducted by feeding back summaries of the interviews when participants indicated that they would appreciate that.

### 2.4 Data-analysis

Data collection and data-analysis was done iteratively and collectively. First, data was discussed in monthly –audio recorded and transcribed verbatim –dialogue meetings with the research team, including the community researchers. Second, the first author conducted an inductive thematic analysis using Maxqda. Third, these findings were validated in the natural group discussion. Fourth, after the thematic analysis, an intersectional perspective was applied to deepen our understanding of the ways initial themes were shaped at the intersections of e.g. gender, class, race, migration status, sexuality, disability and age [24–32, 42]. This round of data-analysis and coding in Maxqda was done by the second author, in close collaboration with the first author. This intersectional analysis enabled a more in-depth understanding about the societal inequalities that became visible in interviewees’ experiences. The outcomes from each round of analysis were discussed with the community researchers, who validated our findings and added insights from their own experiences as well as from the interviews they had conducted. The community researchers were not involved in the writing of this article, as they are not proficient in English. All findings and insights are discussed with them; all (co-authors) consent to the content of this article. The community researchers played a major role in the conception, preparation, data-collection and analysis of this study. Not including them as co-researchers was considered unethical to the academic researchers, particularly in a PHR process [43].

### 2.5 Quality and rigour

In line with our epistemological approach, this study adheres to quality criteria for qualitative research such as credibility, transferability, dependability and confirmability, which are rooted in phenomenological, social constructivist and critical theory [33, 44]. The following strategies were employed to enhance the quality and rigour of our study [44]. The study was conducted with a team of multiple academic and community researchers. The community researchers were experts-by-experience as low-paid care workers in LTC. The academic researchers had expertise in the field of LTC, gender and occupational health. Intensive dialogue about the findings contributed to the credibility of our study. At the request of our participants, member checks on our study findings were conducted by sending a summary of preliminary findings and requesting feedback. Data collection and analysis were done iteratively and impacted following interviews. Data was coded by two researchers (first and second author) supervised by a senior-researcher (last author). Codes were discussed extensively until consensus was reached (researcher triangulation). Interviews were conducted until data saturation was reached, which supports the dependability of our findings. Findings were discussed with societal and LTC organizations in steering group meetings. Their reflections validated our findings, which contributed to the confirmability of our study. Although our study is restricted to residential LTC for older people, our findings contribute to a better understanding of gender in health care workplaces and how these gendered (work) environments affect health. This contributes to the transferability of our findings.

### 2.6 Ethical considerations

This study was evaluated by a Medical Ethical Review Committee which confirmed that the Dutch Medical Research Involving Human Subject Act did not apply (dd. April 17th, 2018). Transcripts were anonymized and audio-tapes and transcripts are stored anonymously and will be archived until five years after completion of the study. Participants signed informed consent forms which are securely stored.

## 3 Results

Our empirical findings are described in four themes. First, ‘*You have to get sick first*’, addresses the normative ideas that shape the ideal care worker including the taboo on self-care. Second, ‘*They won*’*t say to us: go home and get some rest*’ describes the lack of societal and organizational care for LTC workers. Third, ‘*We just take care of each other*’, shows how care workers respond to this lack of care by employing relational health strategies, which render them depend upon colleagues to protect, maintain and repair their health. Fourth, “*Some just want to stick together*”, describes how these relational health strategies can contribute to in- and exclusion within care teams.

### 3.1 ‘You have to get sick first, before you can start taking care of yourself’

This theme addresses the normative ideas that shape the ideal care worker. Interviews showed how many participants foreground their ‘caring identities’ as a prerequisite for being a good care worker. This caring identity is often performed through self-sacrifice and silencing one’s own body:

‘*My mother likes clean curtains, so if she asks me to come, I won*’*t say no. And at the end of the day, you are in pain and then don*’*t even dare to say so. (..) So, yes, I go on even if I am in pain.*’ *(R3)*

In practice, several participants use painkillers, to literally silence their own bodies. Many describe how self-care is not just out-of-character, but rather a taboo. Self-care is not socially accepted within care settings, as it would compromise their caring identity. Participants describe how self-care only becomes legitimate after having compromised one’s health at work, both physically and mentally, for example with burn-out:

‘*You have to get sick first, before you can start taking care of yourself.*’ (R5)

### 3.2 ‘They won’t say to us, go home and get some rest’

Many participants in our sample feel uncared for by society and LTC organizations, translating into feelings of pain, anger and frustration. Care organizations’ focus on production and quality of care comes at the expense of care workers’ health and caring responsibilities. Most care workers understand these problems as structural, and describe how ‘good’ managers have to deal with shortages of personnel and that a large ‘span-of-control’ makes it impossible to take care of their team:

‘*Even if we would all be working seven days a week, we still couldn*’*t fulfill all shifts. Do you understand? So, the manager won*’*t see to us, just go home and get some rest*’ *(R10)*

Several care workers critique these larger structures and express hope that care workers will organize themselves to critique this lack of appreciation. At the same time, many care workers can hardly envision a different (political) reality, compromising their hope for change:

‘*I: Does it make you angry? R: No, I don*’*t get angry. I do my work with love, so, no I don*’*t get angry. I: But would you like it to be different? R: Well, they won*’*t allow it to be different because the insurance company won*’*t pay for it. I: Do you think it would be a good idea if the insurance companies would pay for it? R: Yes, but I don*’*t think so* ...   *well, that won*’*t happen. I: but would you like it to happen? R: definitely, because the people we care for really need it*’. (R2)

### 3.3 “We just take care of each other”: relational health strategies

As a result of the lack of societal and organizational care, care workers turn to each other to protect, support and maintain their health. This relational health strategy is a response to the political and organizational context. Lacking hope for political change, they consider it their individual responsibility to find solutions *within* this system:

‘*I have a great sense of duty, and loyalty to the organization that I work for, and my team, and if I have the drive to* ...   *I just don*’*t like to quit. So, it puts me in the mode of, where are possibilities to keep going*’ *(R9).*

Their solution to the lack of organizational care and support, is taking care of each other:

‘*If they don*’*t take care of their employees, the employees just have to take care of themselves.*’ *(R19)*

‘*You receive more support from your own colleagues, than from a manager.*’ *(R4)*

Many care workers speak of care teams in terms of a ‘family’ that takes care of each other:

‘*We are just one big family (..) Because we work well together and keep each other safe.*’ *(R5)*

Relational health strategies were strengthened by shared experiences, for example in this team where menopausal transition yielded solidarity among team members:

‘*We take care of it together (* ...  *) We are all going through menopause. So, we all have moments that we just can*’*t do it, and then we are there for each other. That is all really easy and good*’*. (R5)*

### 3.4 “Some just want to stick together”: the flip-side of relational health strategies

This theme describes how these relational health strategies can contribute to exclusion within care teams. Not everyone is equally included in ‘the family’ of a care team. This ‘family atmosphere’ often required the exclusion of those who did not fit:

‘*I: How does this atmosphere came about? R: Well, that took us a long time. We had to throw all the rotten apples out of the basket.*’ *(R8).*

Participants in our sample who were not financially dependent upon their care work, often because they could fall back on their male, high-or-middle classed, partner income, and could afford to work part-time to restore their health or do unpaid care work, narrated more extensively about solidarity within teams. They embodied the ideal worker, and were easily included within the team. This respondent for example shared how not being financially dependent upon your care work made it easier for her to be a ‘good colleague’:

‘*I have a small contract, for 8 hours. (* ...  *) See, if you work 24 hours a week then you have to work. But I can work. If a colleague is sick or has a party to go to, I can just see, no problem, I can take your shift*’*. (R18).*

Those who cannot adhere to the normative expectations of the ‘ideal care worker’, more often experienced exclusion from care teams. Exclusion was shaped by (dis)ability, for example because when participants suffered from health issues and had to set their boundaries (self-care):

‘*You can just handle less. (* ...  *) And some colleagues don*’*t accept it. (* ...  *) For example, when I said, I can*’*t do it, it*’*s just too heavy, they would say: well, just try anyway. (* ...  *) I would be like, come on. Some colleagues really had little sympathy for me.*’ *(R13)*

The normative ideal of ‘being available’ was also reproduced with teams, as a marker of solidarity and involvement. Often, this availability was made possible by heterosexual relationships or not having young children. Women who are not always available, for example single mothers, do not fit the norm and could not always count on sympathy from team members:

“*This is a group of women who would say like* “*Well, I also had to take care of my children when I was your age. (* ...  *). So, I am not going to take her children into account now.*’ *(R8)*

The participants in our study often expressed frustration about the ‘younger generation’ as they were not embodying the norm of self-sacrifice, according to the ‘older generation’:

‘*Those young girls, they come and work with us, but then also want to play sports on Saturday, go to the movies on Sunday. They go to the gym two times a week and out to party one time a week. (..) I would be like, (cynically) you want to combine all of that with working in long-term care?*’ *(R22).*

Women who are facing financial precarious situations, do not fit the norm and were othered. Many care workers, who were not financially dependent, implicitly stated ‘doing it for the money’ compromised women’s caring identity:

‘*They told us:* “*In many nursing homes people work for the money, but here in (name organization), you work for people.*” *To me, that was a great compliment.*’ (R9)

Sameness was also challenged when women who were seen as ‘culturally other’ by the dominant group within a care team, also experienced exclusion:

‘*Always these questions. Oh, you don*’*t drink alcohol? No? I just get sick and tired of it. Also. Also, with Ramadan:* “*You shouldn*’*t do it. It*’*s not healthy*”*. Pfff, just very tiresome.*’ *(R10)*

Those who faced exclusion within care teams, as they could not adhere to the norm, experienced negative health effects as a consequence of these experiences of exclusion, further stressing their health. Who does and does not fit in the team, was distinctly shaped by norms of the ‘ideal worker’, and embraced or pushed out by these relational health strategies:

‘*I can understand if you don*’*t feel well sometimes, and that you share it, so you can take care of each other. But after two to three times, you start thinking* “*hmm*”*. And, after the fourth time, you are like, cursing to yourself, I am not here to do both our jobs. I am already running faster than I can. I know, we cannot all carry the same load, but after a while you just think to yourself, can we please get somebody else to work here?*’ *(participant FG).*

## 4 Discussion

Our empirical findings show that gendered norms limit possibilities for self-care for nurses and nursing aides. Many care workers in our sample felt uncared for by society and LTC organizations, and, as a consequence, turned to each other to protect, repair and maintain their own health. These relational health strategies thrived by a feeling of sameness, limiting space for diversity and disability within care teams. Care workers that were seen as ‘culturally other’, or dealt with (chronic) health issues, financial struggles or informal caring responsibilities, faced exclusion within care teams, negatively impacting their health.

The lack of societal and financial appreciation for care workers in LTC is a persistent societal issue, also in the Netherlands [45, 46]. LTC is at the bottom of the cure-care hierarchy [11, 46]. Feminist political scholars have theorized this as a *gendered* inequity [47]. In our current neoliberal financialized version of capitalism, care is framed as a *costly burden* for society, rather than an *investment.* This discourse allows paid care work to be underpaid and re-allocated to lower professional levels, and from paid to unpaid care workers [19], contributing to the *precarization* of paid care workers [45, 48, 49].

Our empirical findings show that this lack of appreciation is not always politicized –or that political change feels out of reach –by female care workers. In response, they turn to each other to maintain, protect and repair their health. This is a gendered response, connected to their socialization *as women,* before and during their vocational training and working lives [50–53]. Care workers’ relational health strategy can also be understood as a *care ethical* response to the given societal and political context [54]. Care ethics –rooted in feminist thought –critiques individualistic notions of autonomy and emphasizes the interdependency of human beings. Therefore, care ethicists rather speak of ‘relational autonomy’ [54, 55]. This relational autonomy is reflected in female care workers’ strategy to protect their health, not as individuals, but in relation to each other. These relational health strategies of female care workers contrasted with the health strategies of men in LTC, who tend to employ more individualistic strategies in response to the lack of appreciation [39, 56–59]. This illustrates the importance of a gender perspective on occupational health [12]. Therefore, HRM interventions for female care workers should not focus solely on individual health strategies, but take these gendered relational dynamics within care teams into account.

Our empirical findings also illustrate the flip-side of these relational health strategies, as they contribute to in- and exclusion within care teams. These health strategies become mechanisms through which inequalities are (re-)produced within organizations, conceptualized by Acker as ‘inequality regimes’ [32]. This is in line with (critical) scholarship on ‘inclusion’ in organizations, which illuminates that (organizational) attempts of inclusion often result in exclusion, as inclusion is contingent upon dominant norms within organizations and/or communities [60, 61]. Our findings illustrate how normative expectations of ‘the ideal care worker’ shaped care workers experience of being in- or excluded within care teams. Intersectionality helped to unravel how these normative expectations, and thus mechanisms of exclusion, were shaped by gender, class, race, age, life phase and disability.

Our empirical findings also illustrate that in- and exclusion should not be viewed as mutually exclusive practices, but as relational practices that are mutually constitutive and inextricably related [60, 61]. In the field of care ethics, this tension between in- and exclusion is described by Hankivsky who argued that care ethics often tend to focus on care for ‘*close others*’, yet in doing so bears the danger of overlooking societal power structures and mechanisms of in- and exclusion [62].

Exclusion can be understood as horizontal violence, conceptualized as ‘*violence in the form of action, words, and other behaviors that is directed toward one*’*s peers*’ [63]. Studies in the field of nursing argue that horizontal violence is connected to the lack appreciation and care for care workers, increase in work load and understaffing [63–64]. Paid care workers in our study suggest that exclusion is a result of exhaustion, and they express moral stress as they feel unable to act otherwise given their current working conditions [65]. This is supported by studies on organizational inclusion in low-paid work, which show that societal appreciation, healthy and decent working conditions (including material and livelihood security) are ‘silent preconditions’ for inclusion at the workplace [66]. Inclusion does not solely hinge on psychological safety in the workplace, but is connected the broader political context and labor market inequities [47, 66].

Activist-scholar Paolo Freire stated that these structural issues are not made political, it can lead to ‘horizontal violence’ among oppressed groups [67]. According to Freire, horizontal violence can be countered by political action. Occupational health researchers, including HRM managers and occupational health physicians, often experience powerlessness in relation to governmental policies impacting the health of individual care workers. Occupational health researchers can help to make these issues political by addressing these issues to LTC organizations and on a political level. Theories on moral distress suggest that this will not only benefit the health of care workers, but also their own [65], which resonates with studies in occupational health [c.f. 68, 69].

Insights from this study have several implications for practice. First, on the organizational level, our findings can support gender and diversity-sensitive occupational health care and interventions. Occupational health interventions should not focus solely on individual health strategies, but take relational dynamics within care teams into account. Acknowledging experiences of exclusion based on gender, class, life-phase, race of disability can enhance occupational health care and support a good client-professional-relationship. Second, LTC organizations can include anti-discrimination in their risk inventory and evaluation (RI&E) instruments and/or in interventions such as a preventive medical consultation. And last, but not least, occupational health scholars can join political efforts to address the structural devaluation of LTC, pressuring the health and wellbeing of LTC workers. In the Netherlands, health care professionals are putting structural health inequalities on the political agenda, arguing that (health) care workers are increasingly confronted with health issues that are the direct result of poverty, livelihood and labor-market insecurity. This requires a political solution, as these root causes cannot be solved in the consultation room alone [70].

Future lines of research should include critical perspectives on in- and exclusion in low-wage labor context, and the role of livelihood security (physical and material safety) as pre-conditions for inclusion in the workplace [66]. Findings from this study also describe the added value of intersectionality for occupational health [24]. Intersectionality can advance diversity-sensitive occupational health research and care, but so far studies are still limited. Interviews for this study have been conducted pre-pandemic: but the dynamics in our paper have been exacerbated in corona times. In the Netherlands, the LTC sector was at the bottom of the cure-care hierarchy, and lacked political and societal attention in the first waves of the corona crisis [46]. Studies done among care workers during the pandemic suggest that these mechanisms of in- and exclusion within care teams became more pressing, but more evidence is needed to support this claim.

## 5 Conclusion

The empirical findings from this study provide openings for gender and diversity-sensitive occupational health care in LTC, by taking women’s relational health strategies into account. At the same time, HRM professionals and occupational health physicians should be aware of the flip-side of these strategies, as they contribute to exclusion at the workplace. This dynamic develops in response to the lack of societal, political and financial appreciation of LTC, translating into adverse working conditions for LTC workers. Physical and material safety are necessary preconditions for inclusion in the workplace. Occupational health professionals and scholars should not solely find solutions in the consultation room, or in the workplace, but also address the root causes on a political level.

## Ethical approval

This study was evaluated and approved by the Medical Ethical Review Committee of the Amsterdam UMC (FWA00017598), which confirmed that the Dutch Medical Research Involving Human Subject Act did not apply (#2018.125). Informed Consent has been obtained from all participants.

## Conflict of interest

The authors declare that they have no conflict of interest.

## Supplementary Material

Appendix 1

Appendix 2
