# Supplementary material for: “We just take care after each other”: Relational health strategies of nurses and nursing aides working in residential long-term care as a mechanism of in- and exclusion in care teams
Source: Work. 2024 Aug 6;78(4):983–94. doi: 10.3233/WOR-220653 (PMC11307021; doi:10.3233/WOR-220653)

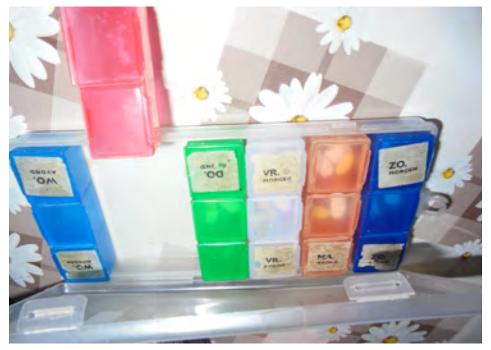
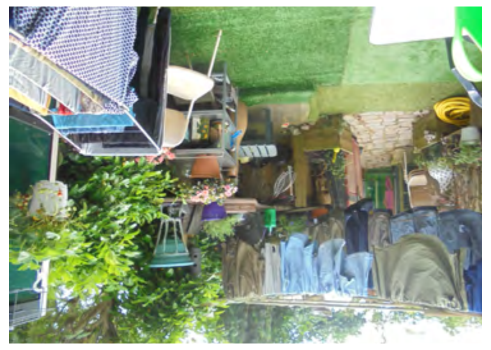
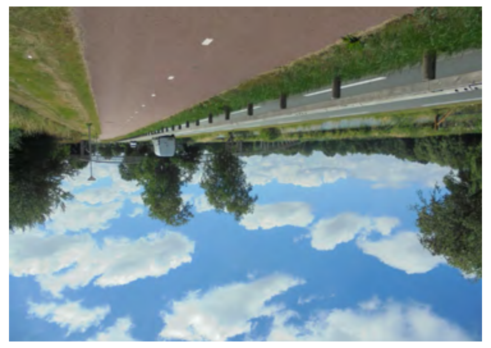
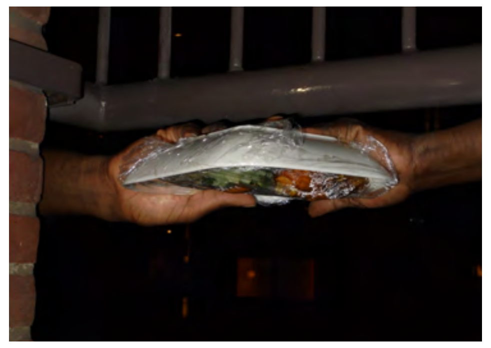
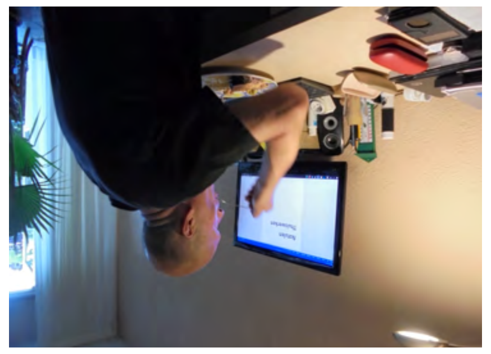
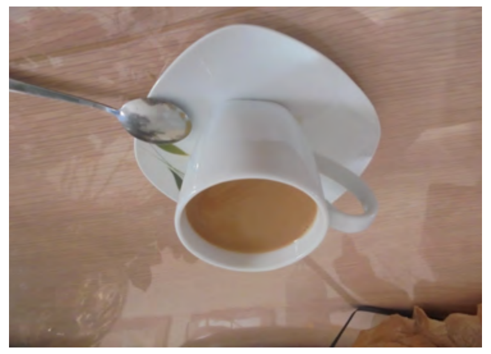
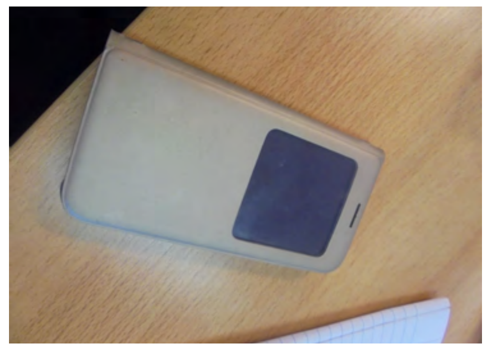
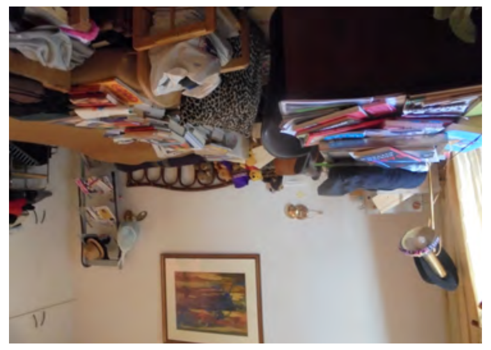

"De afwas laten staan. Gewoon alles in de bak.
Nu ff niet. Het is het einde van de dag.
Het geeft me rust. Nu even tijd voor mezelf.
Ik doe het morgen wel. Maar ja, morgen ben je ook moe.
Er komt geen einde aan. Je laat het liggen.
Maar het moet toch een keer gebeuren."

Zes maanden lang hebben mantelzorgers, zorgmedewerkers en vrijwilligers in de ouderenzorg hun leven gefotografeerd onder leiding van fotografe Janine Schrijver. Aan de hand van deze foto's hebben ze hun eigen leven onderzocht en hebben ze thema's geagendeerd die voor hen belangrijk zijn. De foto's en de geagendeerde thema's ontvouwen zich in deze poster.

Dit **PhotoVoice** project vormt de eerste fase van een participatief actieonderzoek naar de gezondheid van zorgmedewerkers, vrijwilligers en mantelzorgers in de ouderenzorg (45–67 jaar). In de tweede fase van het onderzoek gaan de deelnemers aan het **PhotoVoice** project zelf collega zorgmedewerkers, mantelzorgers en vrijwilligers interviewen. Dat doen ze aan de hand van deze poster. Zie voor meer informatie over het onderzoek **www.vumc.nl/gezond.zorgen**.

Dit onderzoek (2018-2022) wordt gefinancierd door ZonMw in het kader van het programma Gender & Gezondheid. Het wordt uitgevoerd door de afdeling Metamedica van het Amsterdam UMC, locatie VUmc, in samenwerking met Nisa for Nisa, stichting NOOM, FNV vrouw, Cordaan, Markant, FNV Zorg en Welzijn, V&VN, NVAB, ministerie van OCW en Women Inc.

Amsterdam UMC Universitair Medische Centra · FNV vrouw · NOOM · cordaan · Markant · FNV Zorg & Welzijn · Ministerie van Onderwijs, Cultuur en Wetenschap

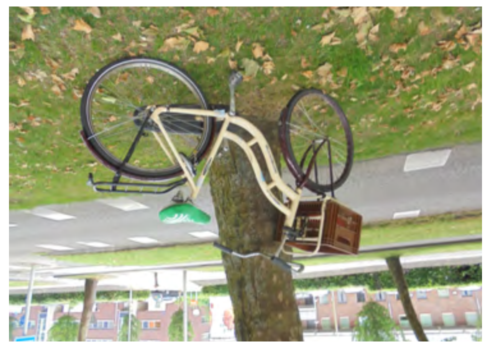
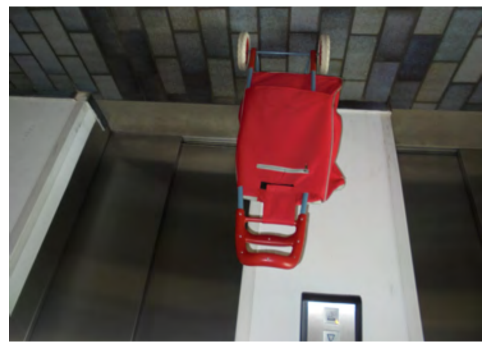

PhotoVoice

Tijdens **PhotoVoice** fotograferen deelnemers hun eigen leven.
De foto's leveren stof op tot gesprek.
De volgende vragen staan daarbij centraal:

Wat **zie** ik?

Wat **gebeurt** hier?

Hoe **raakt** dit aan mijn eigen leven?

Waarom is het zoals het is?

Zou ik willen dat het **anders** was?

Hoe kunnen we het **veranderen**?

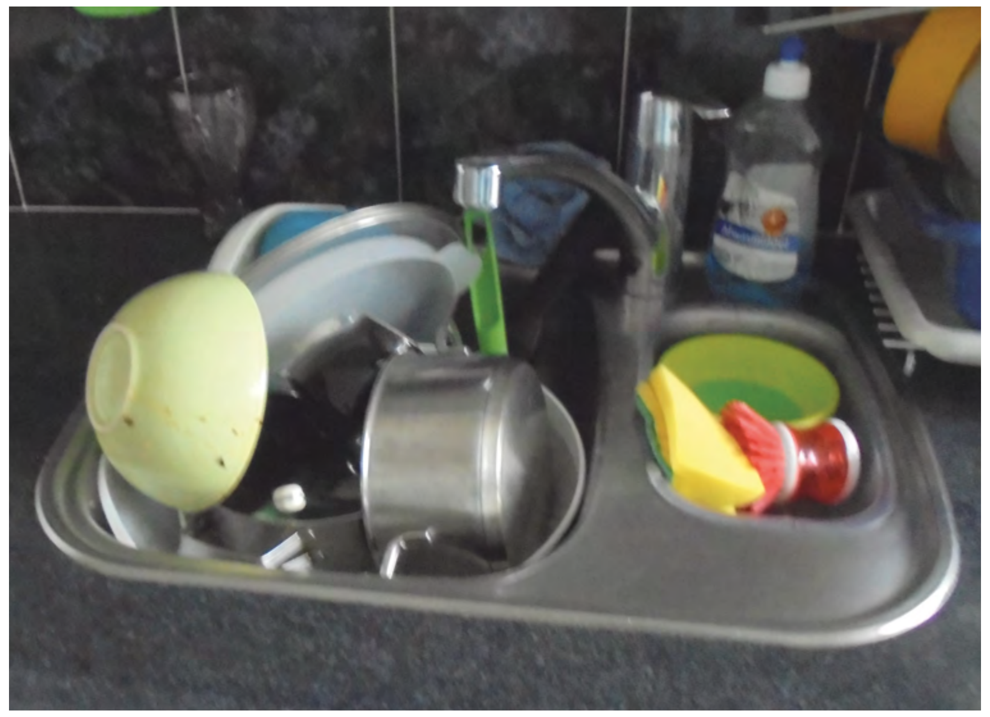

Gezond zorgen

Een participatief actieonderzoek naar
de gezondheid van zorgmedewerkers, vrijwilligers
en mantelzorgers in de ouderenzorg

Dit is mijn verhaal
Wat is jouw verhaal?

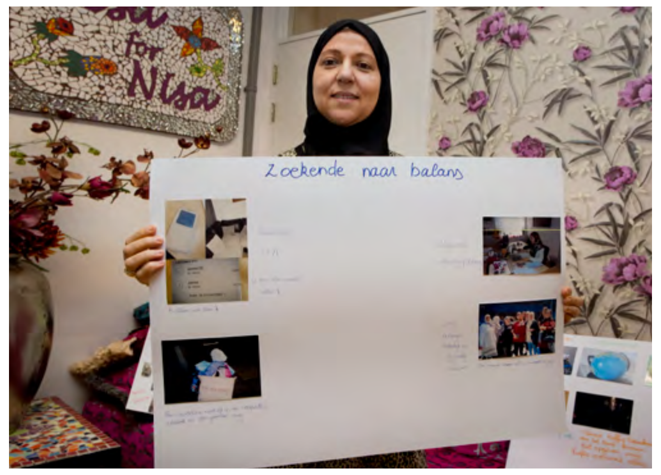

Mensen komen met hun zwaarte bij mij. Die drukt op mij. Ik moet zoeken naar balans, anders raak ik overbelast.

Naziha, 51 jaar
Medewerker in zorg en welzijn, mantelzorger voor haar zusje, vader en tante, vrijwilliger in de buurt

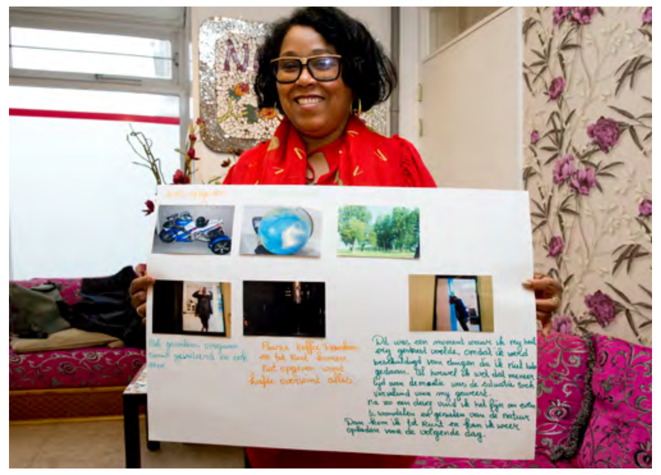

Niet opgeven. Liefde overwint alles. Soms ben ik geïrriteerd of moe. Je moet dan zelf de keuze maken om positief te blijven. Even weglopen en tot rust komen, zodat je de ander weer met geduld en liefde kunt behandelen.

Jachmine, 57 jaar
Mantelzorger voor haar man en buurtvrijwilliger

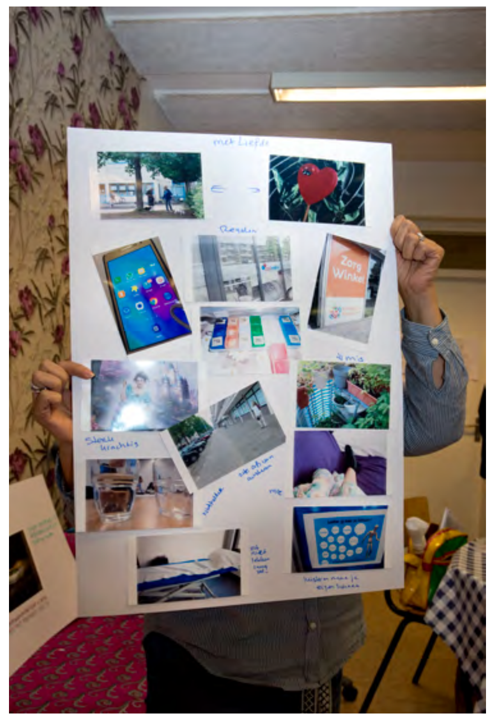

Vroeger was ik sterk en krachtig. Nu ben ik moe. Ik zit niet lekker in mijn vel. Ik zie alles van de achterkant.

Gulay, 45 jaar
Mantelzorger voor haar ouders

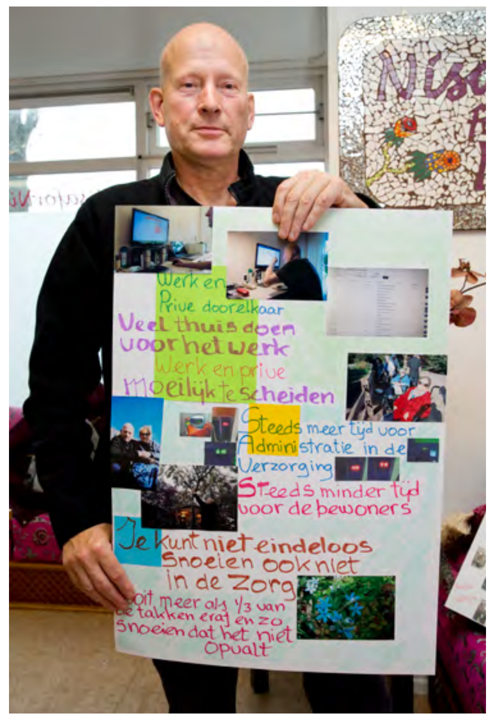

Je kunt niet eindeloos snoeien, ook niet in de zorg.

Max, 60 jaar
Gediplomeerd verzorgende, nu activiteitenbegeleider, is jarenlang mantelzorger voor zijn moeder geweest

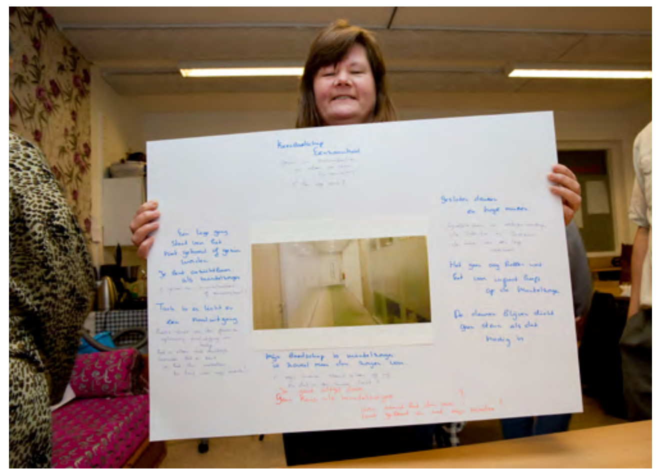

Eenzaamheid. Onzichtbaar zijn. Je staat er alleen voor.

Yvonne, 51 jaar
Gastvrouw in de zorg en mantelzorger voor haar moeder

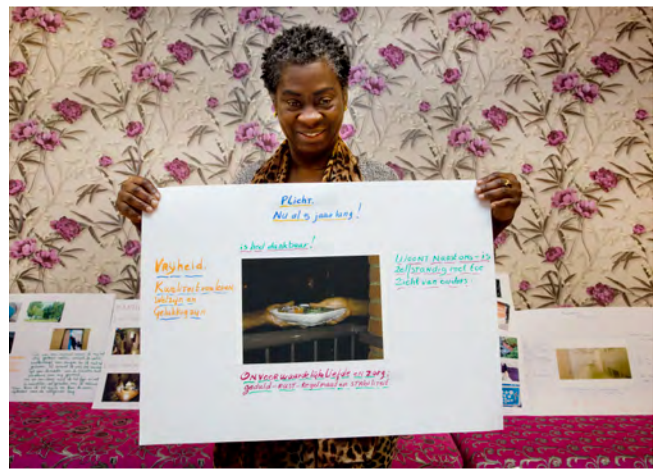

Onvoorwaardelijke liefde, zorg en plicht.

Claudette, 59 jaar
Verzorgende IG en mantelzorger voor haar zoon

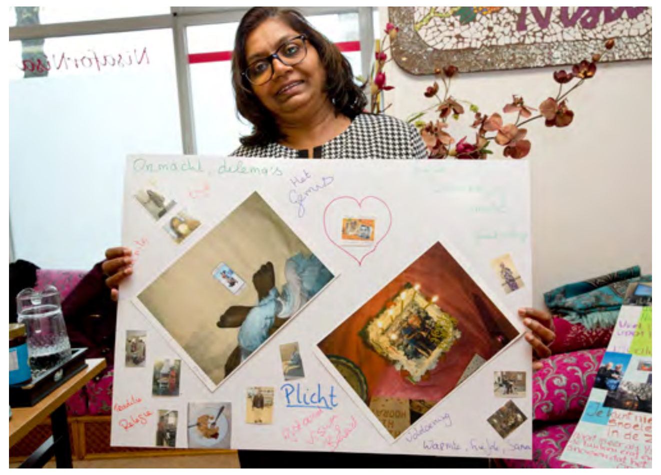

Warmte, liefde, traditie, religie, voldoening van het samenzijn als ze hier is. Maar ook het gemis, de dilemma's en de onmacht als ze in Suriname is.

Ushi, 50 jaar
Verpleegkundige IG, mantelzorger van moeder in Suriname

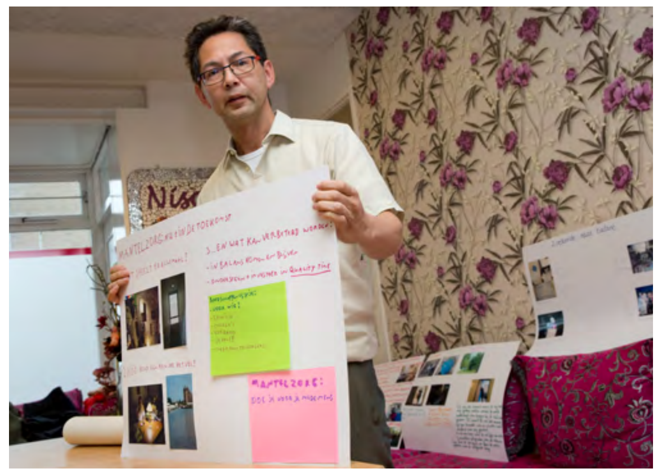

Hoe houdt een kanjer het vol? Zorg in balans. Balans in de zorg.

Piet, 53 jaar
Werkt in de ICT en is mantelzorger voor zijn schoonmoeder

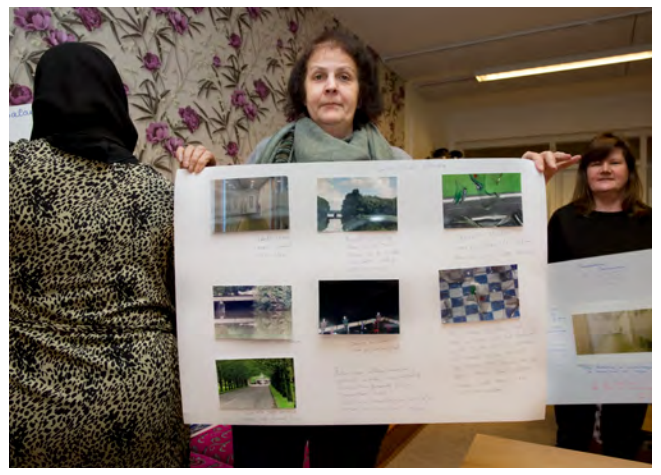

De bewolking staat voor hoe je leven eruit ziet. Je moet zorgen dat je wolken open breken zodat je weer lucht krijgt.

Farida, 52 jaar
Zorgassistente, mantelzorgervoor haar moeder en zorgvrijwilliger

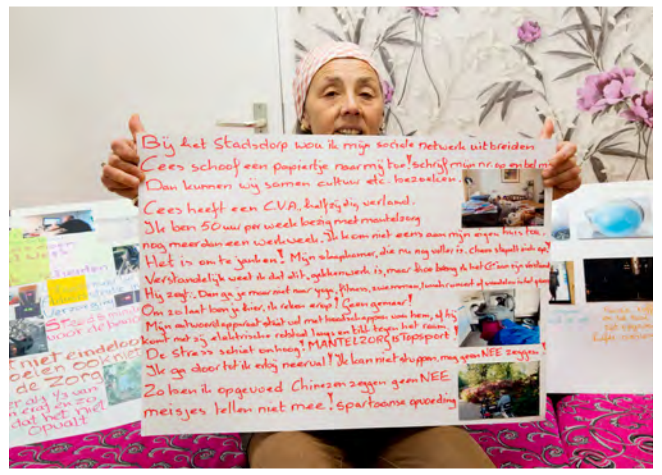

Een grote chaos in het huis en in mijn hoofd. Ik vlucht weg van mijzelf.

Annabel, 62 jaar
Was 35 jaar lang bejaarden-verzorgster, was mantelzorger voor haar moeder, is nu intensieve mantelzorger voor een buurtgenoot

Mantelzorger zijn

Waarom zorg ik?

Zorgwerk en mantelzorg combineren

Vanzelfsprekend werk

"Je rolt erin. Dan komt het moment dat iemand afhankelijk van je is geworden. Dan zit je vast. Je kan iemand niet in de steek laten en zeggen: bekijk het maar."

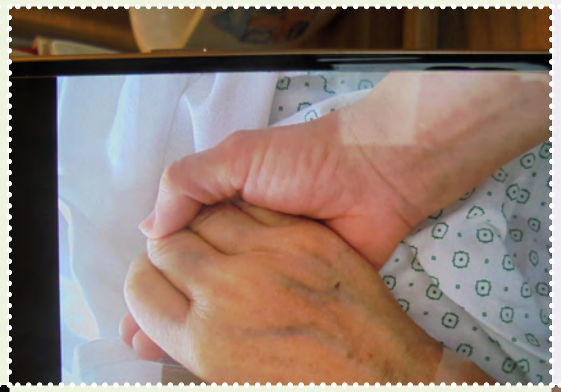

Graag willen zorgen

"Normaal zou ik de plantjes op het balkon van mijn ouders gewoon een plens water geven. Maar nu verzorg ik ze heel precies. Het is alsof ik toch voor mijn moeder kan zorgen, nu ze er niet is."

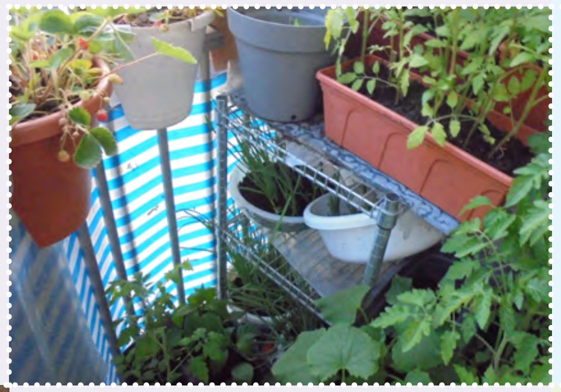

Het zorgen gaat altijd door

"Op mijn werk zorg ik. Thuis zorg ik. Het zorgen gaat altijd door.'

Zwijgen door tijdelijk contract

"Als je een tijdelijk contract hebt dan vertel je niets. Pas als je een vast contract hebt kun je zeggen: ik ben mantelzorger en dat kost me veel energie.'

Kwetsbaar opstellenn

"Als je je kwetsbaar opstelt door je leeftijd, of omdat je mantelzorger bent, word je al snel gezien als ongeschikt. Zit je wel op de goede plek? Kan je het wel aan? Als je zorgt, kun je niet werken. Daar komt het op neer. Dat gevoel krijg je.'

Een risico zijn

"Ze dekken zich in. Je bent risicovol. Het kan tegen je gebruikt worden. Je krijgt een stempel: die zal wel vaak ziekmelden.'

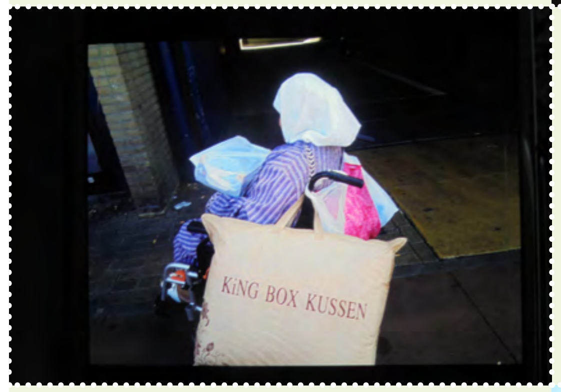

Grenzeloos werk

"Ik ging 's ochtends even met haar wandelen. Toen bleek er veel meer nodig te zijn. Een korte wandeling die 6 uur later eindigde ineen volgeladen rolstoel en een gebroken rug..."

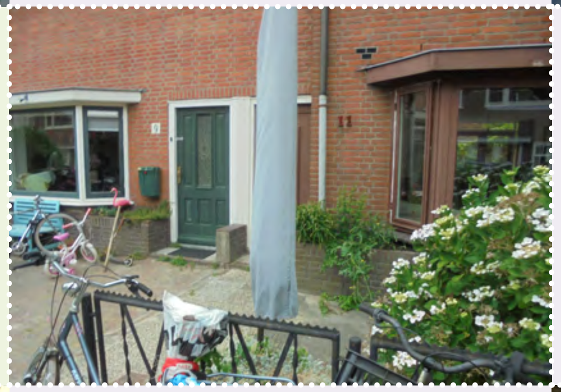

Burenplicht

"Het is gewoon de bedoeling dat je in je buurt een beetje met elkaar optrekt. Dit is toch eigenlijk de burenhulp zoals dat tegenwoordig van je verwacht wordt?"

Werken in de ouderenzorg

Mijn gezondheid

Dankbaar werk

"Als ik iets voor hem doe zegt hij: 'dankjewel hoor mama'. Dan krijgt ik tranen in mijn ogen en denk ik: 'je hoeft mij niet te bedanken, ik ben je moeder.'

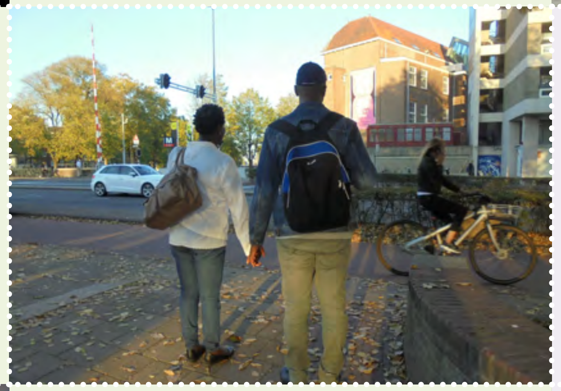

Professionals doen een beroep op mij

"Je wilt graag helpen, maar vervolgens is iedereen allang blij dat jij het doet. De huisarts en andere professionals zeggen: 'hier, doe jij maar'."

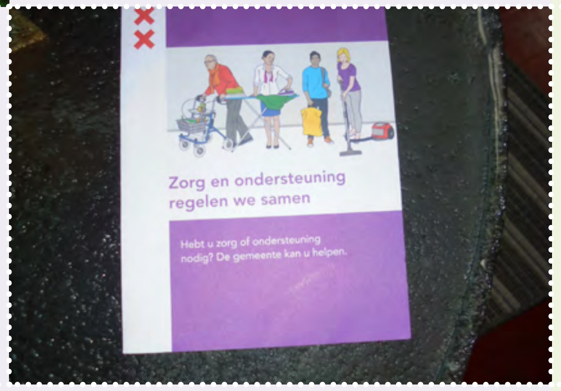

Hoge werkdruk

"Het werk is eigenlijk niet goed te doen en familie en cliënten worden vaak boos op ons. Degene die er het minst aan kunnen doen, worden er het sterkt op afgerekend."

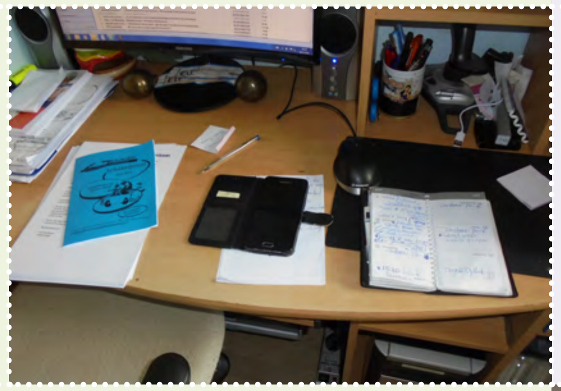

Zorgen voor jezelf

"Ik denk altijd eerst na over de ander. Mijn eigen leven is één grote chaos. Ik vlucht weg voor mijzelf."

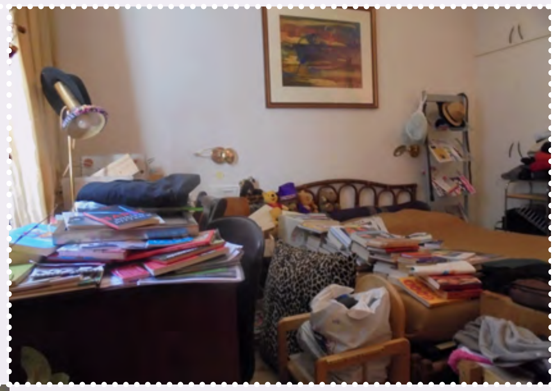

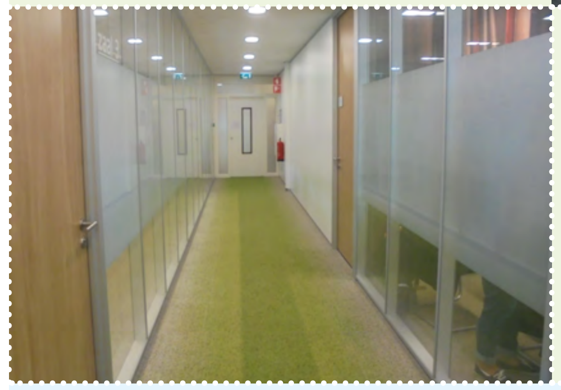

Onzichtbaar werk

"Als mantelzorger ben je vaak onzichtbaar. Je bent niet altijd in beeld bij de zorgverleners. Ik wordt niet gezien als mantelzorger. Soms besef je het zelf ook niet eens."

Onvoorwaardelijke liefde, zorg en plicht

"Als moeder kun je toch het beste voor je kind zorgen. En ja, ik zie het als mijn plicht."

Als formele zorg tekort schiet

"De zorg is gewoon niet goed. Ik kan dat niet aanzien. De verpleging vindt het wel heel makkelijk hè? Zij hebben zoiets van: 'oh zij is er, die helpt je wel'."

Medewerker verantwoordelijk

"In de zorg heb je altijd te maken met onverwachte dingen, maar in een team-overleg werd dat gewoon opzij geschoven en gezegd: 'ja, maar jij moet beter leren plannen'."

Weinig inspraak

"We hebben nu wéér een nieuw systeem gekregen voor de administratie. En waarom? Ons wordt niets gevraagd. Maar we moeten er wel mee werken."

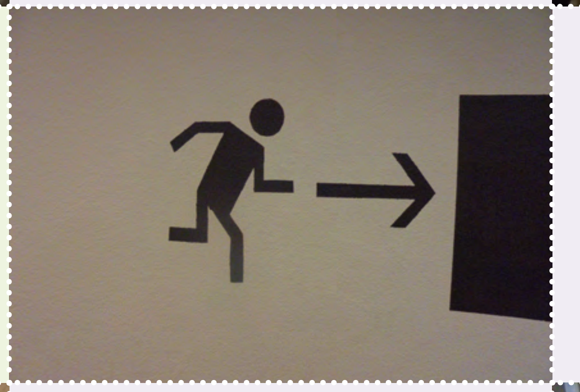

Zorgwerk niet vol te houden tot pensioen

"De verzorging wordt lichamelijk te zwaar. Ik pleeg nu roofbouw op mijn lichaam. Als het lichter werk is, dan zou ik wel langer door willen werken."

Overleven in de ouderenzorg

Minder werken

"Ik ben minder gaan werken. Ik heb echt nee moeten leren zeggen."

Afstompen

"Collega's die te lang in de zorg werken zonder waardering raken ongemotiveerd. Dat heeft effect op de bewoners en op collega's. Solidariteit onderling is soms ver te zoeken."

Altijd beschikbaar moeten zijn

"Ik werd gedwongen om avond- en weekenddiensten te werken. Er werd heel makkelijk gezegd: 'jij moet maar privé dingen in orde gaan maken'."

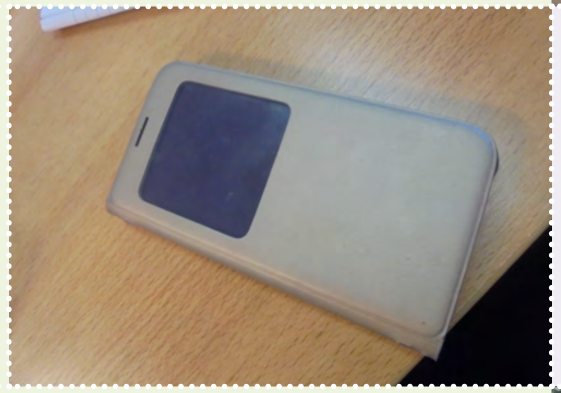

Ziekmelden

"Je meldt je niet zomaar ziek. Want je laat niet alleen je baas in de steek, maar ook de bewoners. Zo voelt dat wel."

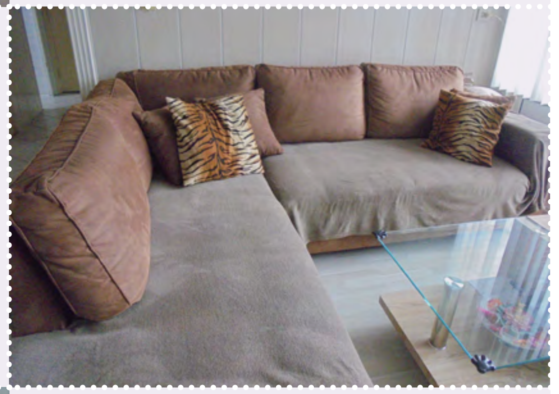

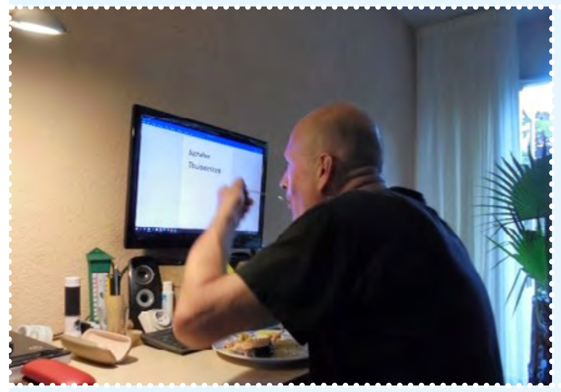

Thuis werken

"Ik zit nog best vaak thuis, als ik vrij ben, achter de computer. Een halve dag ofzo. Op die manier kan ik het aardig redden."

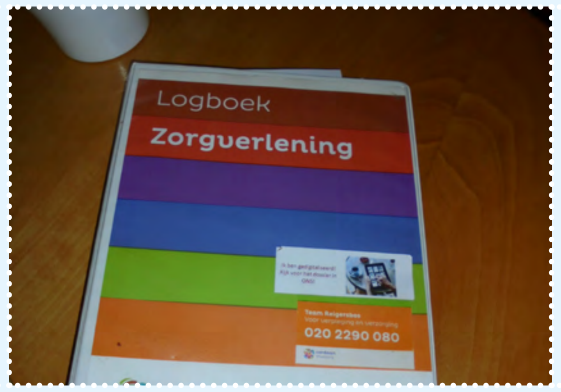

Verzet tegen de administratielast

"Ik vink eerst mijn lijstjes af, daarna kijk ik hoever ik kom."

Moeilijk rondkomen

"Die korte diensten zijn een gedrocht. In feite ben je twee uur per dag werkeloos. Een heleboel hebben nog een baan ernaast om het inkomen op peil te houden."

Gebrek aan waardering

"Ik voel vaak dat mijn werk niet als volwaardig wordt gezien. Alsof het geen écht werk is. Je zorgt voor een medemens en dan wordt je niet gewaardeerd. Dat doet wel pijn."

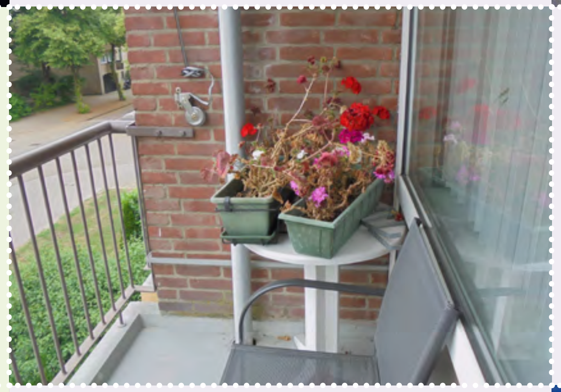

Positief blijven

"Ik maak heel bewust een keuze in mijn leven om positief te blijven."

Onbetaalde overuren

"Ik bleef vaker langer werken, zodat mijn collega's geen last ondervonden. Die uren krijg ik niet betaald. Ze zeggen: 'jij moet leren om op tijd naar huis te gaan'."

Iets extra's doen

"Ik doe altijd meer dan van me verwacht wordt. Ik doe mijn werk met liefde. Ik wil doen wat ik zelf goed acht."

Kiezen voor flexwerk of ZZP-schap

"Ik heb ervoor gekozen om te gaan flexen. Ik wil weer zelf de regie hebben over wanneer ik werk en wanneer niet. Ik heb nu minder administratie, minder taken eromheen."

Verzet tegen de regels

"Als ik me aan alle regels zou houden, dan zou ik mijn werk nooit goed kunnen doen."

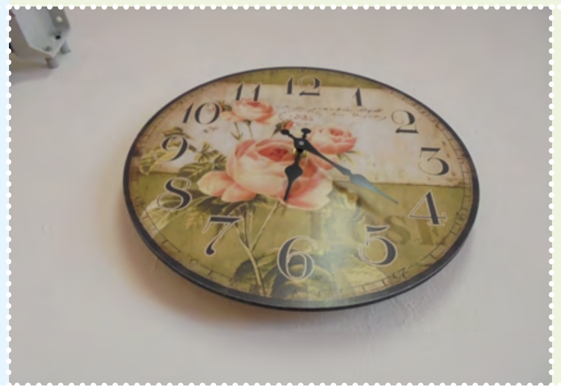

Wisselende diensten

"Juist die wisselende diensten maakt

het werk zo pittig. Bij vaste diensten kun je wat makkelijker de dingen regelen."

De overgang

"Door de overgang ben ik minder belastbaar. Dat merk ik wel. Daar spreek ik niet over op mijn werk."

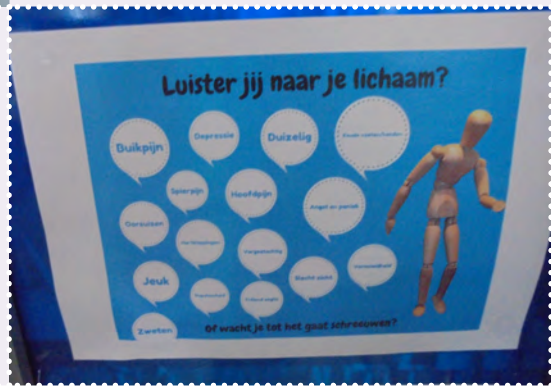

Supplement: Appendix 1 [file wor-78-wor220653-s001.pdf]
