# Supplementary material for: “We just take care after each other”: Relational health strategies of nurses and nursing aides working in residential long-term care as a mechanism of in- and exclusion in care teams
Source: Work. 2024 Aug 6;78(4):983–94. doi: 10.3233/WOR-220653 (PMC11307021; doi:10.3233/WOR-220653)
Supplement: Appendix 2 [file wor-78-wor220653-s002.docx]

[Title of research blinded for review]

# Interview vragen

**PRAKTISCHE ZAKEN**

- Kort informatie over het onderzoek geven
  - Doel van het onderzoek
  - Wat gebeurt ermee
- Jezelf voorstellen
- Poster toelichten: samen hebben we deze thema’s op de agenda gezet
- Informed Consent tekenen
  - Vertrouwelijkheid benadrukken
- Toestemming vragen om het gesprek op te nemen?

**START VAN HET GESPREK**

- Zou je iets over jezelf willen vertellen?
- U heeft de poster ontvangen met daarop de thema’s die voor ons belangrijk waren.
  - Welke thema’s spreken je/u het meeste aan?
  - Waar zou je/u het over willen hebben?
  - Herken je een van de uitspraken op de poster?
  - In welke uitspraken herken je jezelf juist helemaal niet?

**LEVENSLOOP**

- Waarom ben je ooit in de ouderenzorg gaan werken?
- Carrière: aaneengesloten carrière vs. onderbroken carrière (zij-instromer, eruit geweest, switchen).
- Impact van ontslagrondes/bezuinigingen, specifiek rond 2012-2015

**WERKEN IN DE OUDERENZORG**

- Werkdruk? En vooral: impact op eigen gezondheid?
- Grenzen stellen aan werkuren versus grenzeloze appèl van werkgever om rooster rond te krijgen (overwerk, extra dage, etc).
  - Ziek zijn in eigen tijd?
  - Reactie van collega’s als je grenzen stelt (o.a. bij ziekte, of werken in ‘eigen tempo’).
- Kunt u rondkomen van uw inkomen? Is het financieel mogelijk om minder te werken? (kostwinnerschap?)
- Ervaart u steun van de leidinggevenden rondom de werkdruk?

**DE PLICHT OM TE ZORGEN**

- Socialisatie: wat heb je meegekregen over je verantwoordelijkheid om te zorgen?
- Voel je plicht/verantwoordelijkheid om je buren te helpen?
- Ervaar je dat professionals een (groter) beroep op je doen in de zorg?
- Voel je de plicht om in te springen als je ziet dat de zorg tekort schiet?
- Herken je het gevoel dat je altijd blijft zorgen, altijd wilt zorgen?
- Voel je je altijd verantwoordelijk om te zorgen? Hoe komt dat?

**MANTELZORGER ZIJN**

- Mantelzorg
  - Wie, waarom, met wie, ervaring.
- Ervaring met grenzen stellen als mantelzorger?
- Rol van werk in grenzen stellen (hebben van werk is een excuus)
- Combineren werk en & mantelzorg: wat voelt zwaarder?
- Begrip werkgever?
- Levensverhaal: jonge mantelzorger geweest? Leven lang zorgen? (KOPP-kind?)

**ZEGGENSCHAP**

- Opleiding in hiërarchische cultuur: niet tegen arts mogen spreken, etc.
- Ervaren verschil in zeggenschap tussen: man-vrouw, generaties, culturele achtergrond, zwart-wit.

**DISCRMINATIE/UITSLUITING**

- Ervaringen op de werkvoer (van collega’s, van patiënten, van naasten)
- Coping (zwijgen/spreken & rol generaties)
- Minder werken? Twee keer zo hard werken
- Ervaring met diversiteit op de werkvloer (kliekjes? Frustraties onderling?)

**OVERLEVEN IN DE OUDERENZORG**

- Werkdruk: Hoe zorgt u ervoor dat uw werk en andere taken toch gedaan krijgt?
- Werk je (vaak) over?
- Worden je overuren betaald?
- Zou u, of kan u, nog iets spontaan of extra’s doen voor de bewoners of voor uzelf?
- Wat vindt u onrechtvaardig aan de manier waarop de zorg nu is ingericht?
- Wordt je weleens boos over hoe de dingen gaan op de werkvloer?
- Hoe verzet u zich tegen de dingen die u onrechtvaardig vindt?
- Hoe verzet u zich tegen de vastgestelde regels in de zorg?
- Hoe gaat u om met uw emoties?
- Wat doet u om positief te blijven?

**WERK EN ZORG COMBINEREN (thema overbelasting?)**

- Waar loop je tegenaan in het combineren van werk en mantelzorg?
- Hoe houdt je het vol?
- Wat geeft je energie?
- Heb je het gevoel dat je vastloopt? Kun je hier wat meer over vertellen?
- Wat zou een oplossing kunnen zijn om het te kunnen combineren?
- Vertel je het op je werk? Waarom wel/waarom niet?
- Hoe ervaar je dat er door collega’s en leidinggevenden op gereageerd word?
- Is het anders wanneer je een vast contract hebt dan wanneer je een tijdelijk contract hebt?

**MIJN GEZONDHEID**

- Is deze baan vol te houden tot aan je pensioen?
- Wat doe je om gezond te blijven? Herken je je in: grenzeloos werken, dan crisis (burn-out/overgang) en dan minder of geheel de zorg uit? Eigenlijk geen ruimte voor gezondheidsstrategie: of alles geven, of eruit stappen?
- Hoe zie je het voor je tot aan je pensioen? Denk je daar al weleens over na?
- Voel je je gewaardeerd?
  - Zo ja, door wie?
  - Krijg je steun van partner en kinderen bv. of delen jullie taken?
  - Wie zorgt er voor jou als jij ziek bent?
- Wat doe je als het je teveel wordt?
- Welke dingen (taken/ klusjes / bezigheden) zou je kunnen laten?
- Welke dingen kun je niet loslaten?

**OVERGANG**

- Ik ervaar nu ik in de overgang ben dat het me soms teveel wordt (weer zelfde klachten burn-out).
  - Merk jij of heb jij iets gemerkt van de overgang?
  - Zo ja, wat betekende dat voor je werk?
  - Hoe uitte zich dat en kon je dat woorden geven en met wie wel/niet bespreken?
  - Was er ruimte en begrip voor? Hoe zag dat eruit?
- Verwarring overgang-burn-out
- Overgang als bezinningsfase:
  - Andere keuzes gemaakt in werk?
  - Lichamelijk: meer klachten, minder fit
  - Waardering van eigen kennis (erkenning van eigen kennis, willen doorgeven van eigen kennis, niet meer ‘gekoeieneerd’ willen worden van bovenaf)
  - Bezinning:
    - wil ik dit nog? Wie ben ik, wat vindt ik belangrijk?
    - Jezelf voorop gaan stellen: wat wil ik eigenlijk?
    - Keuze om uit de zorg te gaan? Als ZZP’er verder te gaan?

**AFSLUITING**

- Wilt u nog iets toevoegen?
- Kent u nog anderen die mee zouden willen doen met dit onderzoek?
- Mogen we u uitnodigen voor een bijeenkomst waarin we de uitkomsten van het onderzoek presenteren?
- Overhandig de VVV-bon
